# Amine-Modified Carbon Xerogels as Effective Carbon-Based Adsorbents of Anionic Dye from Aqueous Solutions

**DOI:** 10.3390/ma15165736

**Published:** 2022-08-19

**Authors:** Magdalena Ptaszkowska-Koniarz, Joanna Goscianska, Aleksandra Bazan-Wozniak, Robert Pietrzak

**Affiliations:** Faculty of Chemistry, Adam Mickiewicz University in Poznań, Uniwersytetu Poznańskiego 8, 61-614 Poznań, Poland

**Keywords:** carbon materials, amine functionalization, thymol blue, Langmuir isotherm, pseudo-second-order kinetic model

## Abstract

Carbon xerogels were obtained by polycondensation of resorcinol and formaldehyde in a water medium. Their surface was oxidized by ammonium persulfate and then modified with amine groups. Four amines were used: methylamine, ethylamine, propylamine, and ethylenediamine, differing in carbon chain length and number of amine groups. The materials were characterized by low-temperature nitrogen sorption, elemental analysis, thermal analysis, X-ray diffraction, infrared spectroscopy, and determination of the surface oxygen group content with the use of the Boehm method. The final carbon adsorbents had surface areas ranging from 172–663 m^2^/g and acid–base nature. They were applied for adsorption of thymol blue from water solution. The sorption capacities of the studied adsorbents ranged from 83 to 140 mg/g. The presence of amine groups on the xerogel surface was found to increase its sorption capacity towards the dye studied. The dye adsorption process is endothermic and spontaneous, as indicated by the positive values of ΔH and the negative values of ΔG, respectively. The kinetics of adsorption of thymol blue was established to be described by the pseudo-second-order model. The equilibrium data were analyzed by the Langmuir and Freundlich models. The character of thymol blue adsorption is much better described by the Langmuir isotherm.

## 1. Introduction

Water, in addition to oxygen and nutrients, is necessary to sustain life. Its appreciation increases with the development of urbanization, industry, and agriculture. Water pollution has become a problem of top priority on a global scale. The most troublesome pollutants include: dyes, nitrates (V), phosphates (V), chlorates, sulfates (V), heavy metal ions, aromatic amines, pesticides, detergents, and bacteria. The largest amounts of toxic pollutants are introduced to the water systems with wastewater. Other important sources are water and land transport, pesticides and artificial fertilizers used in agriculture, and municipal and industrial waste [1,2,3]. All water pollutants are hazardous to human health, so maintenance of water purity is a priority among environmental protection issues. Appropriate measures should be taken to limit the amount of pollutants entering the natural water system, as well as to effectively remove already-introduced contamination. The latter is realized through wastewater purification. Depending on the type of pollutants, different methods are applied: mechanical, biological, physical, and chemical. As follows from the literature, many methods of water treatment have been proposed to deal with the problem, including: ion-exchange, coagulation, reversed osmosis, ozonation, electrochemical methods, adsorption, and chemical precipitation [4,5,6,7,8,9]. In order to remove pollutants from post-industrial wastewater by sorption methods, it is necessary to apply effective adsorbents [10,11,12], represented by, e.g., carbon xerogels, characterized by well-developed surface area and offering a possibility of easy modulation of pore size distribution [13]. According to the literature, they have been successfully used for sorption of orange II [14], chromotrope 2R [14], methylene blue [15], and rhodamine B [16]. Satisfactory results, obtained using carbon xerogels as adsorbents towards cationic dyes, have been reported by Girgis et al. [17]. The high sorption capacities of carbon xerogels, which increased with extended internal porosity, were comparable with those of conventional activated carbons. Figueiredo’s group [18] analyzed the sorption capacities of carbon xerogels oxidized by nitric (V) acid towards anionic dyes. According to the data, the sorption capacities of these adsorbents were comparable to those of commercial activated carbons, in the same conditions of the process, and ranged from approximately 90 to 160 mg/g.

**Figure 1:**
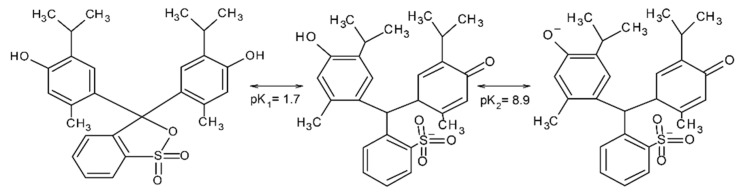
Structures of thymol blue at different pH.

The aim of our research was to synthesize carbon xerogels modified with different amines, perform comprehensive characterization of them, and test their performance as adsorbents of thymol blue from water solutions. Amine groups, as strong electron donors, enhance the basic character of the carbon xerogels surface, so their presence was expected to facilitate the adsorption of thymol blue, which is an anionic dye [16]. An important task was to establish the effect of pH of the adsorbate solutions and temperature of the process on the efficiency of adsorption of carbon xerogels. Thymol blue is a chemical pH indicator, as it changes colors from red to yellow in the pH range 1.2–2.8 and from yellow to blue in the pH range 8.0–9.6 [19,20,21]. The two ranges of the color changes of this indicator are related to the two stages of its dissociation (Figure 1).

## 2. Materials and Methods

### 2.1. Samples

Carbon xerogel (CX) was obtained as follows: 0.040 L of distilled water was added to 25 g of resorcinol (Merck SA, Darmstadt, Germany) while stirring [22]. When the resorcinol was dissolved, a solution of NaOH (2 mol/L, Chempur, Piekary Śląskie, Poland) was added in order to bring the pH to 5.3. Then, 34 mL of formaldehyde (Chempur, Piekary Śląskie, Poland) was added while stirring, and the pH was adjusted to 5.3 by adding a solution of HCl (0.1 mol/L, Avantor Performance Materials Poland S.A., Gliwice, Poland). The gelation was accomplished in a water bath over three days (85 °C). The gel was crushed and dried in an oven for 4 days. The dried gel was carbonized at 700 °C in a tubular furnace (N_2_—150 mL × min^−1^, heating rate—2 °C × min^−1^). Pyrolysis was realized in the following stages: (1) up to 200 °C, held for 2 h; (2) up to 300 °C, held for 1 h; (3) up to 700 °C, held for 2 h. The carbon xerogel obtained was oxidized by 1 mol/L solution of (NH_4_)_2_S_2_O_8_ (Merck SA, Darmstadt, Germany) in a 2 mol/L solution of H_2_SO_4_ for 6 h (30 °C)—the obtained adsorbent was denoted by the symbol CX-APS. At subsequent stages, the sample CX-APS was modified by methylamine (MA), ethylamine (EA), propylamine (PA), and ethylenediamine (EDA) (Merck SA, Darmstadt, Germany). For the modification, 2 g of each amine was mixed with 30 g of methanol (Avantor Performance Materials Poland S.A., 99.8%, Gliwice, Poland), and, after 30 min, 2 g of the adsorbent CX-APS was added to each mixture; the mixtures were stirred at 40 °C for 6 h. Then, the mixtures were filtered off, and the samples were washed with methanol and dried at 70 °C. The carbon xerogels functionalized with amines were labelled as CX-APS-MA, CX-APS-EA, CX-APS-PA, and CX-APS-EDA.

### 2.2. Samples Characterization

#### 2.2.1. XRD

The samples CX, CX-APS, and CX-APS-EDA were characterized by powder X-ray diffraction in the wide-angle range using a D8 Advance Diffractometer (Bruker, Billerica, MA, USA, CuK_α_ radiation, λ = 0.154 nm, step size 0.05°).

#### 2.2.2. Nitrogen Sorption, Elemental Analysis and Acid–Base Properties

Elemental analyses of the samples were performed with the use of a Vario ELIII elemental analyzer (Elementar Analysen systeme GmbH, Langenselbold, Germany). The textural parameters of the obtained adsorbents were characterized on the basis of low-temperature nitrogen adsorption/desorption, using an Autosorb iQ instrument, provided by Quantachrome Instruments (Boynton Beach, FL, USA). Before adsorption measurements, the samples were degassed under vacuum for 8 h, at 200 °C. The surface areas of the samples (S_BET_) were evaluated in the range of relative pressure p/p_0_ from 0.05 to 0.30 (according to the Brunauer–Emmett–Teller method). The total pore volume (V) of each sample was estimated based on the amount of liquid nitrogen adsorbed at a relative pressure p/p_0_ = 0.99. The mean pore diameter (D) was calculated from the equation D = 4 V/S_BET_. Moreover, the commonly known t-plot method was applied to determine the micropore volume and area. The content of the surface oxygen functional groups, both acidic and basic, was determined by standard neutralization titration, with HCl and NaOH, according to the Boehm method [16,23,24].

#### 2.2.3. Thermogravimetric Analysis and Infrared Spectroscopy

Thermal stabilities of the samples were measured on a Setsys 1200 Setaram (Setaram, Lyon, France). The measurements were performed under flowing nitrogen at a heating rate of 10 °C/min, over a temperature range of 25−1000 °C, with an initial material weight of approximately 10 mg. Structural changes in the carbon xerogel, after modification with amine groups, were detected by FT-IR spectroscopy. Prior to measurement, the samples were mixed with dried potassium bromide at a rate of 0.5 mg of the adsorbents to 200 mg of KBr. The spectra, in the range of 400−4000 cm^−1^, were recorded on an FT-IR spectrometer Bruker IFS 66v/S (Billerica, MA, USA) [24].

### 2.3. Adsorption of Dyes

Portions of 20 mg of each sample were added to 50 mL of thymol blue solutions (concentrations range of dye: 12.5–150 mg/L). The flooded samples were stirred by a magnetic stirrer for 1 day (temperature 22 ± 1 °C). Then, the samples were filtered off and the absorbance of the filtrate was measured by a UV-Vis spectrophotometer, model Carry 100 Bio (Agilent, Santa Clara, CA, USA). The measurement was made twice at λ_max_ = 435 nm. On the basis of the standard curve, the final concentrations of the dye were determined and the sorption capacity of each sample was calculated. The amount (q_e_) of thymol blue adsorbed on a given carbon adsorbent, expressed in mg/g, was calculated from formula (1):

(1) $$q_{e}=\frac{C_{0}-C_{e}}{m}\cdot V$$

where *C*_0_ is the initial concentration of thymol blue (mg/L), *C_e_* is the residual concentration of thymol blue (mg/L), *V* is the volume of thymol blue (L), and *m* is the mass of carbon xerogel adsorbent (g).

The effect of the pH of the thymol blue solution on the sorption capacities of the carbon xerogel adsorbents was determined, as the pH values varied in the range of 2–12 (pH-meter ELEMETRON, Zabrze, Poland). The pH of each solution was adjusted with 0.1 M HCl and 0.1 M NaOH solution. Measurements were performed for 20 mg of the carbon xerogel adsorbents and 50 mL of the dye solution, with 80 mg/L concentration.

The adsorption isotherms were fitted to the two models proposed by Langmuir and Freundlich. The Langmuir adsorption isotherm [25] is described by the following linear Equation (2):

(2) $$\frac{C_{e}}{q_{e}}=\frac{1}{K_{L}\times q_{max}}+\frac{C_{e}}{q_{max}}$$

where *q_e_* is the experimentally determined amount of the thymol blue adsorbed on the carbon samples (mg/g), *q_m_* is the theoretically predicted amount of the adsorbed dye (mg/g), *C_e_* is the equilibrium concentration of the dye solution (mg/L), and *K_L_* is the Langmuir constant.

The linear form of Freundlich isotherm equation [16] is expressed as (3):

(3) $$lnq_{e}=lnK_{F}+\frac{1}{n}lnC_{e}$$

where *q_e_* is the experimentally determined amount of the thymol blue adsorbed on the carbon samples (mg/g), *C_e_* is the equilibrium concentration of the dye solution (mg/L), and *K_F_* and 1/*n* are the Freundlich constants, characteristic of a given system.

The value of 1/*n* (varying from 0 to 1) is a measure of the intensity of adsorption or heterogeneity of the surface. The closer it is to zero, the more heterogeneous the surface [16].

In order to check the effect of contact time on the effectiveness of the adsorption of the dye studied, 20 mg portions of each sample were added to 50 mL of thymol blue solutions (150 mg/L). The samples were stirred by a magnetic stirrer. At selected time intervals (10, 20, 30, 40, 50, 60, 120, 150, 180, 240, 300, 360, and 1440 min), the absorbance of the dye solution was measured. Kinetics of the dye adsorption on carbon xerogels was characterized on the basis of comparison to two kinetic models: pseudo-first-order (4) and pseudo-second-order (5):

(4) $$\ln\left(q_{e}-q_{t}\right)=lnq_{e}-\frac{k_{1}t}{2.303}$$

(5) $$\frac{t}{q_{t}}=\frac{1}{k_{2}{q_{e}}^{2}}+\frac{t}{q_{e}}$$

where *q_t_* is the amount of the dye adsorbed on the surface of carbon xerogels in a given time (mg/g), *k*_1_ is the adsorption constant in the pseudo-first-order equation (1/min), and *k*_2_ is the adsorption constant in the pseudo-second-order equation (g/mg × min) [6].

Sorption studies were also carried out at different temperatures (25, 45, and 60 °C). The procedure of sample preparation was the same as was used when studying the effect of the pH of the dye solution on the adsorption capacities of the carbon adsorbents. The thermodynamics of the thymol blue adsorption on the carbon xerogels adsorbents was characterized based on the enthalpy, entropy, and Gibbs free energy, calculated from the formulas described in our work [24].

## 3. Results and Discussion

Results of the elemental analysis of the carbon xerogels, modified with different amine groups, are given in Table 1.

Pure carbon xerogel (CX) and carbon xerogel modified with methylamine (CX-APS-MA), ethylamine (CX-APS-EA), and propylamine (CX-APS-PA) showed elemental carbon content above 70 wt. %. The lowest content of elemental carbon was found in CX-APS. This sample had the highest content of sulfur (19.32 wt. %) and oxygen (44.29 wt. %) among all xerogel samples studied. Pure carbon xerogel did not contain nitrogen and sulfur. The sample modified with ethylenediamine (having two amine groups) was distinguished from the others by higher nitrogen (9.23 wt. %) and hydrogen (3.61 wt. %) contents.

**Figure 2:**
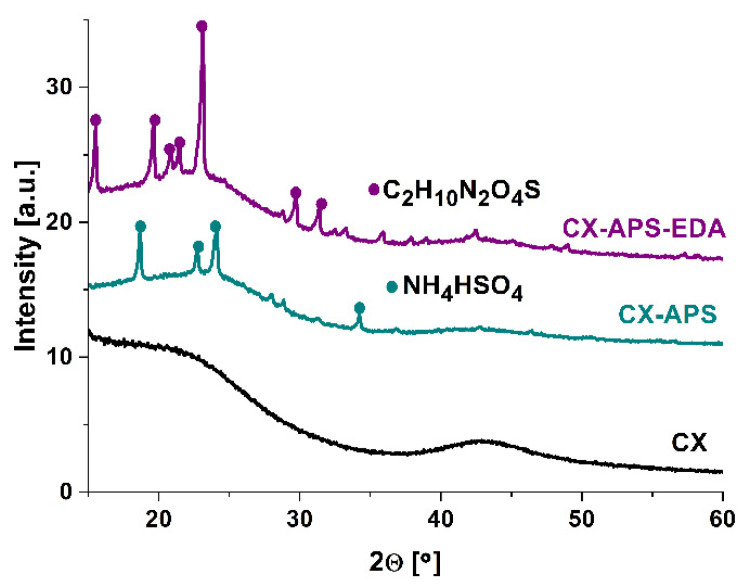
Wide-angle XRD patterns of carbon xerogels: CX, CX-APS, and CX-APS-EDA, recorded at room temperature with a step size of 0.05°.

Figure 2 displays the wide-angle range of XRD profiles of the three samples: CX, CX-APS, and CX-APS-EDA. The diffractogram of CX sample showed no reflections. The XRD profile of the sample CX-APS exhibited peaks assigned to ammonium hydrosulfate [26]. After the modification of CX-APS with ethylenediamine, the XRD profile contained additional peaks, assigned to ethylenediamine sulfate (C_2_H_10_N_2_O_4_S) [27]. In the case of the samples functionalized with the other amines, no reflections were observed.

Table 2 presents the textural parameters of all adsorbents studied. Pure carbon xerogel (CX) was found to have the largest specific surface area and the greatest pore volume. CX oxidation, by ammonium persulfate, led to the reduction of textural parameters: surface area, pore volume, and micropores area. It is supposed that the process of modification takes place mainly inside small mesopores and micropores. As a consequence of this process, oxygen-containing groups may partly block the pores of carbon xerogel, leading to a decrease in the specific surface area of CX-APS [16,27]. Functionalization with amines results in an increase in these parameters, with respect to those of sample CX-APS. This may lead to deblocking of the pores, and thus an increase in the specific surface area and pore volume [16,27]. After the modification of CX-APS with ethylenediamine (CX-APS-EDA), only the pore diameter was reduced (20.46 nm) compared to the pore diameter of CX-APS sample (21.19 nm). Most probably, upon functionalization with ethylenediamine (with two amine groups in a molecule), the xerogel pores are blocked with amine groups.

**Figure 3:**
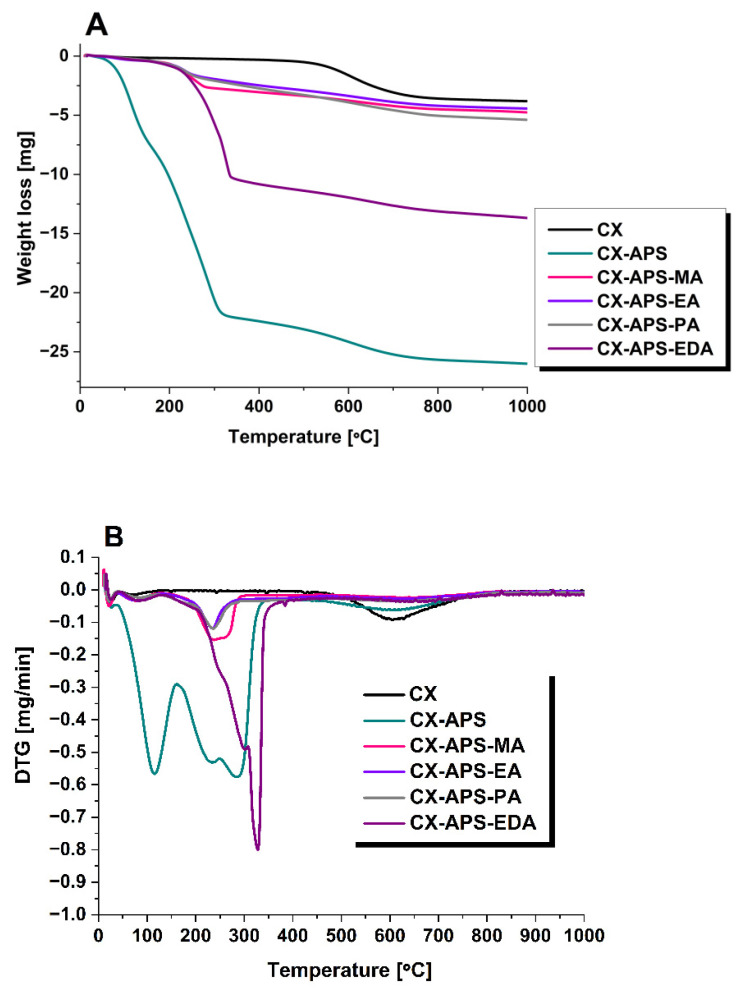
TG (**A**) and DTG (**B**) curves from 25 to 1000 °C of pure and amine-modified carbon xerogels registered under N_2_ atmosphere.

The TG (thermogravimetric) curves obtained upon heating the xerogel samples in a nitrogen atmosphere are depicted in Figure 3A, while Figure 3B presents the DTG (derivative thermogravimetric) curves that illustrate the rate of mass loss with increasing temperature. For pure carbon xerogel, the greatest mass loss was observed in the range of 500–600 °C, which is interpreted as corresponding to the removal of O_2_, H_2_, and CO_2_ (Figure 3) [24]. For sample CX-APS, the mass loss observed was the most pronounced. The mass loss of the CX-APS sample, visible in Figure 3, up to about 100 °C, most probably corresponds to the elimination of the physically adsorbed water. The mass loss noted in the range of 180–300 °C is related to decomposition of carboxylic groups [28,29,30]. The TG and DTG curves of the samples modified with methylamine, ethylamine, and propylamine (CX-APS-MA, CX-APS-EA, CX-APS-PA) have similar smooth courses. The mass loss is visible for them in the range of 50–250 °C; the first takes place up to 100 °C and is ascribed to the elimination of physisorbed water. The mass loss at higher temperatures (180–250 °C) was assigned to the degradation of carboxylic groups. For the sample modified with ethylenediamine (CX-APS-EDA), a significant mass loss was detected at about 330 °C, which most probably corresponds to the decomposition of amine groups (Figure 3).

**Figure 4:**
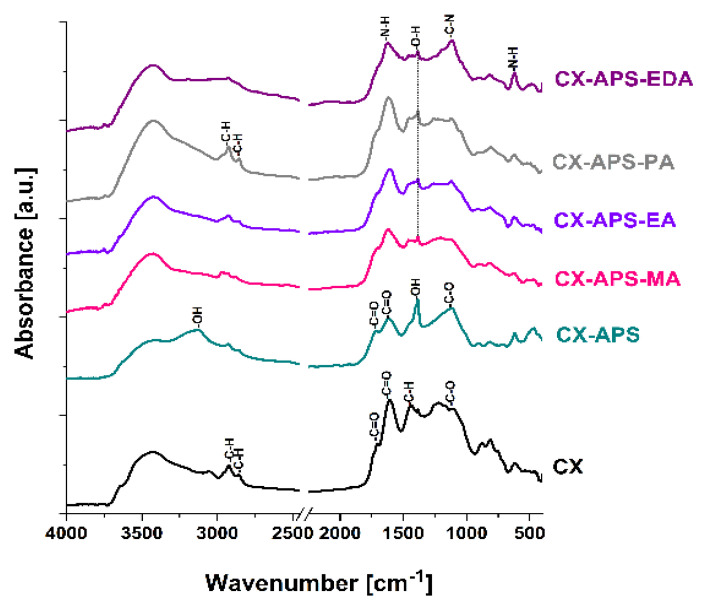
FT-IR spectra of samples studied.

The FT-IR spectrum (Figure 4) of CX sample shows bands at ~2925, 2858, 1441, and 1384 cm^−1^, assigned to the stretching and bending vibrations of C-H. The bands appearing at ~1715 and 1615 cm^−1^, on the basis of the literature, are attributed to the vibrations of C=O [28]. The bands in the range of 1217–1094 cm^−1^ come from the stretching vibrations of C-O. The spectrum of the sample oxidized with ammonium persulfate (CX-APS) contains an additional band with a maximum at 3148 cm^−1^, assigned to the stretching vibrations of O-H. It should be added that the FT-IR spectrum of sample CX-APS, besides the bands at ~1715 and 1615 cm^−1^ assigned to the vibrations of C=O, also shows bands in the ranges of 1450–1381 cm^−1^ and 950–910 cm^−1^, corresponding to the deformational vibrations of O-H. The FT-IR spectra of carbon xerogels modified with amine groups show bands assigned to the deformation vibrations of N-H at 1624 cm^−1^. Subsequent bands, characteristic of amines, appear in the range of 910–665 cm^−1^. Bands corresponding to the stretching vibrations of C–N in aliphatic amines are of medium intensity, and appear in the wavenumber range of 1250–1044 cm^−1^ (Figure 4) [31,32,33].

According to data presented in Table 3, the carbon xerogels differ in acid-base properties. The pure CX sample has both basic and acidic functional oxygen surface groups. Its oxidation leads to a considerable increase in the content of acidic groups (6.16 mmol/g), at the complete disappearance of basic groups. Functionalization of carbon xerogels with amines causes the generation of surface oxygen functional groups of basic nature. Their amount is in the range of 0.50–1.49 mmol/g, depending on the type of amine applied for modification (Table 3).

**Figure 5:**
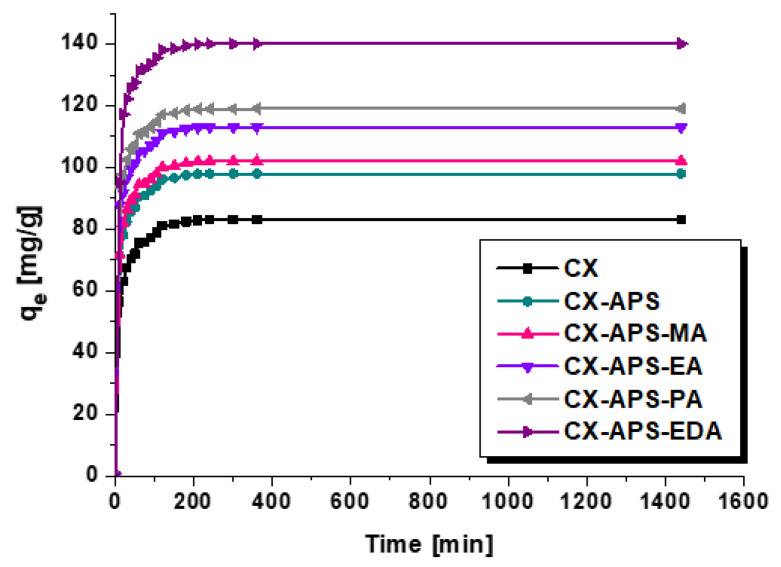
Effect of contact time on the sorption capacity of carbon xerogels (adsorbent mass, 20 mg; initial dye solution concentration, 150 mg/L; volume of dye solution, 50 mL, pH = 4; temperature, 22 ± 1 °C).

Figure 5 illustrates the effect of the contact time on the sorption capacity of the xerogel samples. In the first 40–50 min, the adsorption process is fast, which can be explained by a large number of free adsorption centers available for the colorant [34]. In the time from 60 to about 240 min, adsorption is much slower, which could be explained by the saturation of available adsorption sites. According to the results presented in Figure 5, after about 240–260 min the state of equilibrium is achieved.

The data fitted to the two kinetic models are given in Table 4. Much higher values of R^2^ were obtained for the pseudo-second-order kinetic model, so this model better describes the thymol blue adsorption. Additionally, for all carbon xerogels, the values of q_e(exp)_ are identical to the theoretical values of q_e(cal)_ calculated assuming pseudo-second-order kinetics.

**Figure 6:**
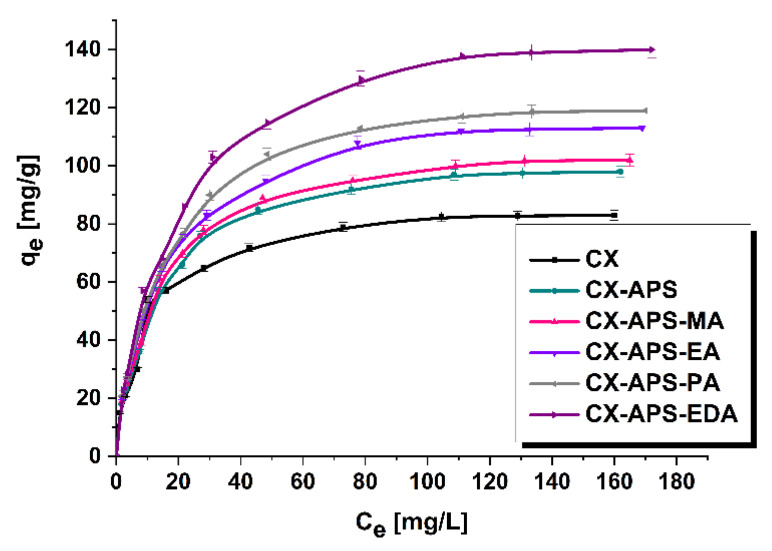
Isotherms of thymol blue adsorption on carbon xerogels (adsorbent mass, 20 mg; initial dye solution concentration, 12.5–150 mg/L; volume of dye solution, 50 mL; pH = 4; temperature, 22 ± 1 °C).

Figure 6 presents the isotherms of thymol blue adsorption on the surface of all samples. The amount of thymol blue adsorbed on the surface of the xerogel samples increases with increasing initial concentrations of the colorant solutions. The amount of thymol blue adsorbed by sample CX (83 mg/g) increases after its oxidation with ammonium sulfate (CX-APS) to 98 mg/g. Moreover, functionalization of CX-APS with amine groups also leads to increased sorption capacity towards thymol blue. Analysis of the results shows that, for CX-APS-MA, CX-APS-EA, and CX-APS-PA, with increasing length of the carbon chain the sorption capacity towards thymol blue increases. However, as the differences in the sorption capacity values are small, it seems that further modification with amines that have longer carbon chains is pointless.

Analysis of the data depicted in Figure 6 leads to the conclusion that the most effective adsorbent is sample CX-APS-EDA, modified with ethylenediamine, which has two amine groups in the molecule, while the other modifying compounds have only one such group. The greatest sorption capacity of CX-APS-EDA, at a level of 140 mg/g, is confirmed by the highest nitrogen content and the basic character of the surface of this adsorbent. The lowest sorption capacity towards thymol blue was found for pure carbon xerogel, containing no nitrogen, which can explain its worse affinity to the dye. Electrostatic attraction and hydrogen bond formation between the colorant molecules and xerogel surface of basic nature are supposed to be the dominant mechanism of adsorption. During the adsorption of thymol blue in a water medium, the hydroxide groups from the dye molecules dissociate. Thus, the sorption capacity of samples towards the dye studied is substantially determined by the interaction of deprotonated hydroxide groups (anions –O-) coming from the dye molecules with the amine groups present on the surface of modified adsorbents [35,36,37,38]. The adsorption of the anionic dye for CX-APS-EDA was enhanced by the presence of a greater number of amine groups on the surface of this sample.

In order to determine the mechanism of thymol blue on the surface of the adsorbents, we used the equations of Langmuir and Freundlich isotherms (Table 5). The value of R^2^ was higher for the fit to the Langmuir isotherm, and ranged from 0.9991 to 0.9994. The data presented in Table 5 are much poorer fitted to the Freundlich isotherm. The values of 1/*n,* for all carbon xerogels, were in the range 0.348–0.409, i.e., lower than 1.

**Figure 7:**
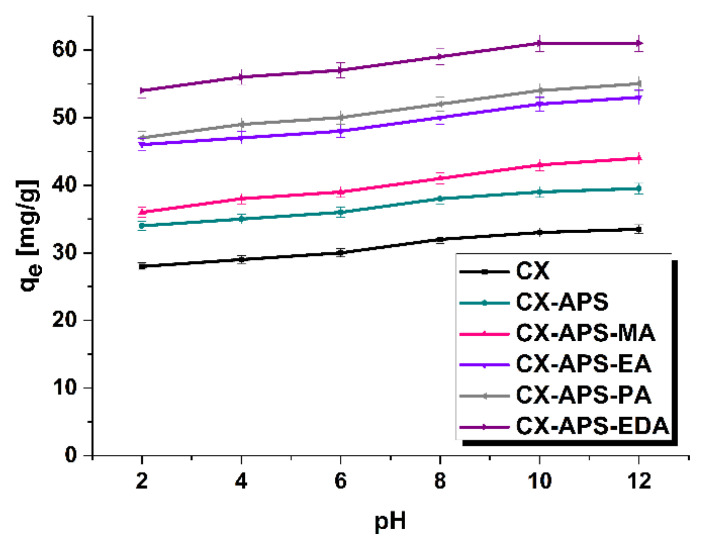
The influence of pH on the adsorption of thymol blue (adsorbent mass, 20 mg; initial dye solution concentration, 80 mg/L; volume of dye solution, 80 mL; temperature, 22 ± 1 °C).

Another problem considered was the influence of the pH of the thymol blue solutions on the sorption capacities of the samples obtained (Figure 7). With increasing pH of these solutions, the amount of the dye adsorbed on the carbon xerogels surfaces increased. This effect was more pronounced for the xerogels functionalized with amines, for which q_e_ increased by about 7–8 mg/g. Thymol blue is negatively charged at pH > 1.7; therefore, the interaction takes place between the sulfonic group or deprotonated hydroxyl group of the dye (Figure 1) and the amine groups, that are usually Lewis bases, present on the xerogels surfaces. This interaction can be stronger in basic environments than in an acidic ones (pH < 7) [39,40,41,42].

**Figure 8:**
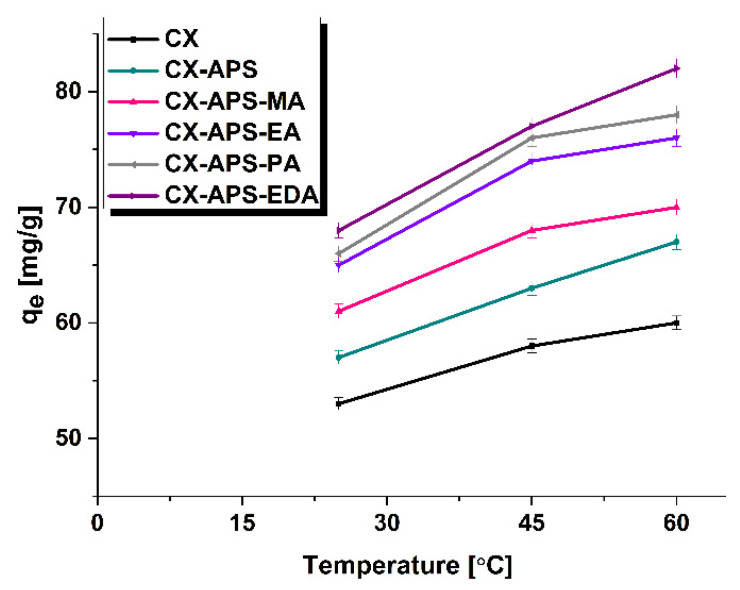
The influence of temperature on the adsorption of thymol blue (adsorbent mass, 20 mg; initial dye solution concentration, 80 mg/L; volume of dye solution, 50 mL; pH = 4; temperature, 25, 45, and 60 ± 1 °C).

The impact of temperature on the sorption capacity of adsorbents towards thymol blue was also studied (Figure 8). According to the results, with increasing temperature, the sorption capacity increased (*q_e_*).

This can be explained by the greater mobility of the dye molecules in the solution, which leads to increased binding of thymol blue molecules to the adsorption sites on carbon xerogels. This effect is the most pronounced for sample CX-APS-EDA, whose sorption capacity increases by 14 mg/g (Figure 8). For pristine xerogel, CX, the increase in q_e_ is the smallest, only by 7 mg/g. Table 6 presents the calculated values of ∆G, ∆H, and ∆S for the adsorption of thymol blue. The positive values of ∆H indicate the endothermic nature of the process, while the negative values of ΔG imply that it is a spontaneous process whose spontaneity increases with increasing temperature [43,44]. The values of entropy vary in the range of 34.60−44.28 J/K mol (Table 6).

Table 7 presents a comparison of the sorption capacities of the CX-APS-EDA sample towards thymol blue, tested in this study, with results obtained for other adsorbents. The comparison implies that CX-APS-EDA is very effective in the removal of thymol blue from water solutions. From the analysis of Table 7 data, it can be observed that the adsorption capacity of the prepared CX-APS-EDA sample is comparable, or superior, to the values obtained in previous studies. Only activated carbon, prepared by chemical activation of garcinia cola nutshell impregnated with H_3_PO_4_, showed higher sorption capacity towards thymol blue.

**Figure 9:**
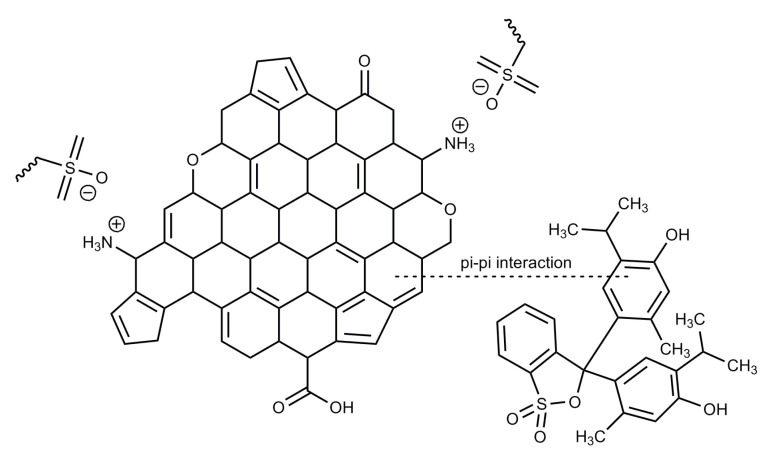
The proposed mechanism of the adsorption of thymol blue onto modified carbon xerogels.

Adsorption of organic dyes may be influenced by the structure of the dye, the properties of the surface of carbon adsorbents, the formation of hydrogen bonds, and electrostatic interactions. The carbon xerogels synthesized in this study have amine groups on their surfaces. Electrostatic interactions are possible between the amine functional groups on the surface of the adsorbents and the sulfonic groups of thymol blue (Figure 9) [40]. The mechanism of adsorption may also involve the formation of hydrogen bonds and π-π interactions.

## 4. Conclusions

Pristine carbon xerogel was subjected to surface oxidation followed by modification with four different amines. The xerogels functionalized with amine groups showed smaller surface areas and pore volumes, but greater average pore diameters, than the unmodified sample. In addition, the sample functionalized with ethylenediamine indicated the highest content of nitrogen and oxygen functional groups compared to the other samples.

The most effective adsorbent of thymol blue from water solutions was the sample modified with ethylenediamine, whose surface exhibited the most basic character of all adsorbents. The value of q_e_ obtained for sample CX-APS-EDA was 140 mg/g. The high sorption capacity of this carbon xerogel was due to its surface properties. The basic surface functional groups enhanced the interaction between the carbon xerogel and the anionic thymol blue. Amine groups (Lewis bases) derived from ethylenediamine sulfate on the surface of carbon adsorbent undergo protonation in an acidic solution of the dye, and therefore may interact electrostatically with the sulfonic groups of thymol blue. In addition, hydrogen bonds and π-π interactions may form during adsorption process.

It was found that the sorption capacities of the samples increased with increasing initial concentration of thymol blue in water solutions. The Langmuir isotherm model and pseudo-second-order kinetic model fitted well to the data of thymol blue adsorption. With increasing temperature, the sorption capacities of the carbon xerogel adsorbents towards the dye studied increased. The thermodynamic analysis suggested that the thymol blue adsorption process was spontaneous and endothermic in nature. Moreover, thymol blue sorption was more effective in a basic environment.

## Figures and Tables

**Table 1:** Elemental analysis of the samples studied (wt. %).

Material	C	H	N	S	O *
CX	78.77	2.60	0.00	0.00	18.63
CX-APS	31.18	3.35	1.86	19.32	44.29
CX-APS-MA	70.97	2.06	2.20	2.79	21.98
CX-APS-EA	77.23	1.96	1.16	1.21	18.44
CX-APS-PA	74.55	2.36	1.26	1.33	20.50
CX-APS-EDA	50.32	3.61	9.23	10.43	26.41

* by difference; method error ≤ 0.3%.

**Table 2:** Textural properties of the samples studied.

Material	Total Surface Area ^1^ (BET) [m^2^/g]	Micropore Area [m^2^/g]	Total Pore Volume [cm^3^/g]	Micropore Volume [cm^3^/g]	Average Pore Diameter [nm]
CX	663	401	1.49	0.171	20.30
CX-APS	155	8	0.82	0.002	21.19
CX-APS-MA	450	229	1.28	0.123	33.65
CX-APS-EA	368	142	1.21	0.075	33.14
CX-APS-PA	340	113	1.14	0.058	26.65
CX-APS-EDA	172	13	0.88	0.005	20.46

^1^ Error range between 2–5%.

**Table 3:** Acid–base properties of carbon xerogels.

Material	Acidic Oxygen Functional Groups [mmol/g]	Basic Oxygen Functional Groups [mmol/g]
CX	1.27 ± 0.02	0.49 ± 0.01
CX-APS	6.16 ± 0.05	0.00 ± 0.00
CX-APS-MA	0.91 ± 0.01	0.50 ± 0.01
CX-APS-EA	1.23 ± 0.02	0.74 ± 0.01
CX-APS-PA	0.91 ± 0.01	0.74 ± 0.01
CX-APS-EDA	0.73 ± 0.01	1.49 ± 0.02

**Table 4:** Kinetic parameters for the adsorption of thymol blue on the surface of carbon xerogels.

Material	q_e(exp)_ [mg/g]	Pseudo-First-Order Model			Pseudo-Second-Order Model		
		q_e(cal)_ [mg/g]	*k*_1_ [1/min]	R^2^	q_e(cal)_ [mg/g]	*k*_2_ [g mg^−1^ × min^−1^]	R^2^
CX	83 ± 1.6	50.90	0.0524	0.9811	83.40	0.0025	0.9999
CX-APS	98 ± 1.9	52.21	0.0533	0.9784	98.43	0.0025	0.9999
CX-APS-MA	102 ± 2.0	54.60	0.0532	0.9707	102.46	0.0025	0.9999
CX-APS-EA	113 ± 2.3	95.64	0.0700	0.9392	113.51	0.0024	0.9999
CX-APS-PA	119 ± 2.4	82.57	0.0817	0.9197	119.47	0.0025	0.9999
CX-APS-EDA	140 ± 2.8	76.30	0.0734	0.9426	140.65	0.0022	0.9999

**Table 5:** The parameters calculated from Langmuir and Freundlich models.

Material	Langmuir			Freundlich		
	q_m_ [mg/g]	*K_L_* [L/mg]	R^2^	*K_F_* [mg/g (L/mg)^1/*n*^]	1/*n*	R^2^
CX	88.2	0.11	0.9991	16.90	0.348	0.8926
CX-APS	105.3	0.09	0.9993	19.16	0.351	0.9280
CX-APS-MA	109.8	0.09	0.9993	18.47	0.367	0.9306
CX-APS-EA	121.9	0.08	0.9994	19.54	0.377	0.9241
CX-APS-PA	128.4	0.08	0.9993	20.07	0.383	0.9188
CX-APS-EDA	153.4	0.07	0.9991	20.51	0.409	0.9332

**Table 6:** Thermodynamical parameters calculated for the adsorption of thymol blue into carbon xerogels.

Material	Temperature [°C]	∆G [kJ/mol]	∆H [kJ/mol]	∆S [J/mol K]
CX	25	−4.13	6.57	35.99
	45	−4.93		
	60	−5.38		
CX-APS	25	−3.57	6.75	34.60
	45	−4.23		
	60	−4.78		
CX-APS-MA	25	−3.65	6.64	34.62
	45	−4.46		
	60	−4.84		
CX-APS-EA	25	−3.71	7.78	38.73
	45	−4.66		
	60	−5.04		
CX-APS-PA	25	−3.72	8.38	40.80
	45	−4.73		
	60	−5.11		
CX-APS-EDA	25	−3.78	9.40	44.28
	45	−4.41		
	60	−4.76		

**Table 7:** Comparison of sorption capacities of the carbon xerogel modified with ethylenediamine to other adsorbents, presented in the literature, towards thymol blue.

Adsorbent	*q_max_* [mg/g]	References
CX-APS-EDA	153.4	This study
activated carbon from *Trachycarpusfortunei* seeds	130.38	[41]
pomegranate peel	5.28	[45]
bentonite	117.6471	[46]
activated carbon from garcinia cola nutshells	396.04	[47]

## Data Availability

Data is contained within the article.
